# Guide Wire Stimulation During Catheter Ablation for Atrial Fibrillation-Induced Cardiac Arrest in a Patient With Preexisting Left Bundle Branch Block: A Case Report

**DOI:** 10.7759/cureus.71949

**Published:** 2024-10-20

**Authors:** Daiki Yamashita, Yoshihiko Kagawa, Masaki Ishiyama, Naoki Fujimoto, Kaoru Dohi

**Affiliations:** 1 Department of Cardiology and Nephrology, Mie University Graduate School of Medicine, Tsu, JPN

**Keywords:** cardiac arrest, catheter ablation, fluoroscopic guide, guide wire, noninvasive transcutaneous pacemaker

## Abstract

Right bundle branch block can occasionally occur when a guide wire or catheter is inserted into the heart. An 83-year-old woman with preexisting left bundle branch block (LBBB) was diagnosed with paroxysmal atrial fibrillation (PAF) and severe mitral regurgitation (MR). The patient was started on amiodarone (100 mg/day) and bisoprolol (1.25 mg/day). The patient underwent catheter ablation for PAF after a percutaneous edge-to-edge mitral valve repair for MR. During the ablation procedure, performed under a sedation with dexmedetomidine, guide wire stimulation led to a paroxysmal atrioventricular block (AVB), resulting in cardiac arrest. Cardiopulmonary resuscitation was performed for 2 min, one ampule of intravenous adrenaline was administered, and a return of spontaneous circulation was obtained. The patient subsequently developed takotsubo cardiomyopathy due to the administration of catecholamines. Three months later, re-ablation was performed safely under fluoroscopic guidance and the use of noninvasive transcutaneous pacemaker. Fluoroscopic guide wire manipulation and the use of noninvasive transcutaneous pacemaker are essential for patients with LBBB to prevent paroxysmal AVB and cardiac arrest.

## Introduction

The incidence of a right bundle branch block (RBBB) during the insertion of guide wires or catheters, such as in central venous catheterization or pacemaker lead placement, is approximately 3-12% [1]. RBBB is generally temporary, with a mean duration of fewer than 24 h, and typically does not require intervention [2]. Patients without a history of left bundle branch block (LBBB) are less likely to experience paroxysmal atrioventricular block (AVB) due to catheter trauma to the right bundle branch (RBB). Guide wire insertion is a crucial step for sheath insertion in catheter ablation, but guide wire stimulation can induce RBBB. Herein, we describe a case of cardiac arrest caused by guide wire stimulation in a patient with preexisting LBBB during catheter ablation.

## Case presentation

**Figure 1:**
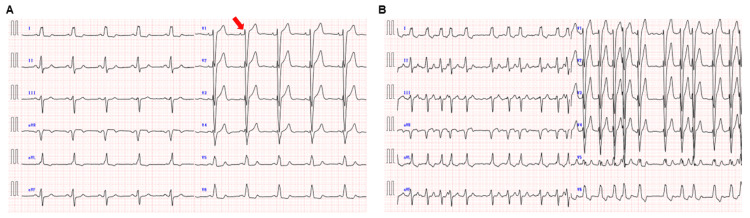
Electrocardiograms ECG showing normal sinus rhythm, LBBB, and an r wave of 4 mm in lead V1 (red arrow, A). PAF occurs frequently after three months following percutaneous edge-to-edge mitral valve repair (B). ECG = electrocardiogram; LBBB = left bundle branch block; PAF = paroxysmal atrial fibrillation

An 83-year-old woman was diagnosed with complete left bundle branch block (CLBBB) during a medical examination nine years ago. The patient presented to a local clinic seven years ago with palpitations and was diagnosed with paroxysmal atrial fibrillation (PAF), leading to the initiation of anticoagulation therapy. Four months ago, the patient visited another local clinic due to persistent palpitations since four days ago and was again diagnosed with PAF and severe mitral regurgitation (MR). The patient was subsequently referred to our hospital for percutaneous edge-to-edge mitral valve repair and catheter ablation. Medications included amiodarone (100 mg/day), bisoprolol (1.25 mg/day), and apixaban (5 mg/day), and medical history was otherwise unremarkable. The patient had no family history of heart disease or sudden death. Laboratory tests revealed an elevated B-type natriuretic peptide (BNP) level of 206.3 pg/mL. An electrocardiogram (ECG) showed sinus rhythm and LBBB (Figure 1A). Transthoracic echocardiography demonstrated normal left ventricular contraction with a left ventricular ejection fraction of 59% and severe MR due to P3 prolapse. Her MR was classified as degenerative MR. Transesophageal echocardiography showed a P3 prolapse due to ruptured chordae. She was initially offered an open heart surgery, but she refused. She preferred a catheter-based operation, percutaneous edge-to-edge mitral valve repair. Three months ago, the patient underwent percutaneous edge-to-edge mitral valve repair, which improved the patient’s severe MR to mild MR. Amiodarone and bisoprolol were continued; however, PAF recurred multiple times after the procedure (Figure 1B). Therefore, catheter ablation was scheduled after obtaining informed consent.

**Figure 2:**
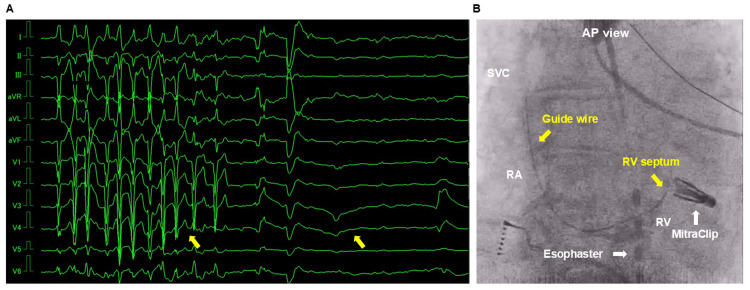
Electrocardiogram and fluoroscopic image PVC and NSVT lead to paroxysmal AVB and cardiac arrest (yellow arrow, A). Fluoroscopic image immediately after cardiac arrest, confirming that the tip of the guide wire was located in the right ventricular basal septum (yellow arrow, B). PVC = premature ventricular contraction; NSVT = non-sustained ventricular tachycardia; AVB = atrioventricular block MitraClip = device for percutaneous edge-to-edge reconstruction of the mitral valve (Abbott, Menlo Park, CA, USA); Esophaster = luminal esophageal temperature monitoring catheter (Japan Lifeline, Tokyo, Japan)

On admission, physical examination revealed a heart rate of 65 bpm, blood pressure of 143/78 mmHg, and oxygen saturation of 97%. Chest X-ray showed a cardiothoracic ratio of 51%. ECG indicated sinus rhythm, a normal PQ interval, LBBB, and an r wave of 4 mm in lead V1. Laboratory tests revealed a BNP level of 29.5 pg/mL, which had decreased compared to the values before percutaneous edge-to-edge mitral valve repair. On the second hospital day, catheter ablation was performed. Dexmedetomidine was started at a rate of 6 µg/kg/min as a loading dose for 10 min. An echo-guided right internal jugular vein puncture was performed, and a guide wire was inserted into the heart without simultaneous fluoroscopic guidance for sheath insertion. Immediately after wire insertion, premature ventricular contractions (PVCs) and non-sustained ventricular tachycardia (NSVT) occurred, followed by paroxysmal AVB and cardiac arrest (Figure 2A). Fluoroscopic imaging immediately after the cardiac arrest confirmed that the tip of the guide wire was located in the right ventricular septum (Figure 2B).

**Figure 3:**
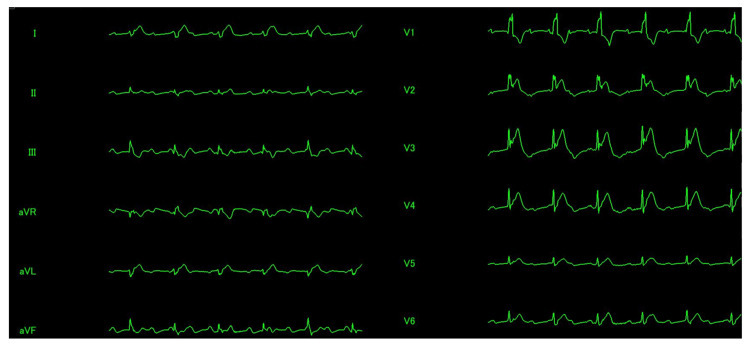
Electrocardiogram ECG showing paroxysmal AVB and ST elevation in leads I, aVL, and V2–V6. ECG = electrocardiogram; AVB = atrioventricular block

Cardiopulmonary resuscitation (CPR) was performed for 2 min, 1 mg of intravenous adrenaline was administered, and a return of spontaneous circulation was obtained. ECG showed paroxysmal AVB and ST elevation in leads Ⅰ, aVL (augmented vector left), and V2-V6 (Figure 3).

**Figure 4:**
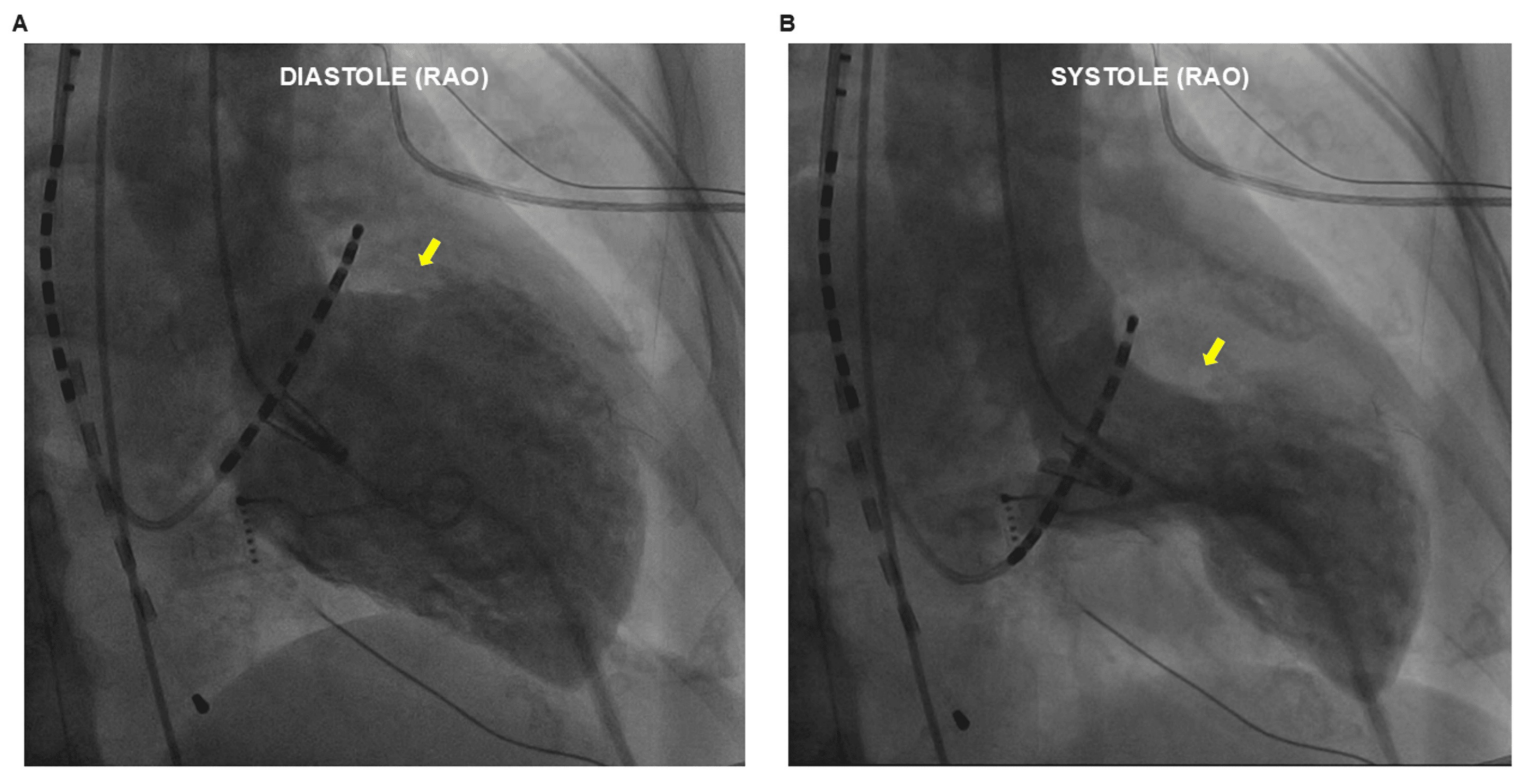
Left ventriculography Left ventriculography revealed akinesis in the apical (segment 3) and hyperkinesis in the basal (segment 1 and 5) segments, confirming a diagnosis of Takotsubo cardiomyopathy (yellow arrow, A: diastole, B: systole) RAO = right anterior oblique

Coronary angiography was performed in an emergency just after AVB occurrence and revealed no significant stenosis. Left ventriculography was also performed because there was no evidence of coronary artery stenosis and the possibility of Takotsubo cardiomyopathy was considered based on ECG changes. Left ventriculography revealed akinesis in the apical (segment 3) and hyperkinesis in the basal (segments 1 and 5) segments, confirming a diagnosis of Takotsubo cardiomyopathy (Figure 4). Hemodynamic status was maintained, and the patient was fitted with an external pacemaker. AV conduction improved during the operation, but the patient was admitted to the intensive care unit with an external pacemaker implanted as a precaution. On the third hospital day, ECG showed giant negative T waves (not shown), which gradually resolved. The patient was discharged home on day 12.

**Figure 5:**
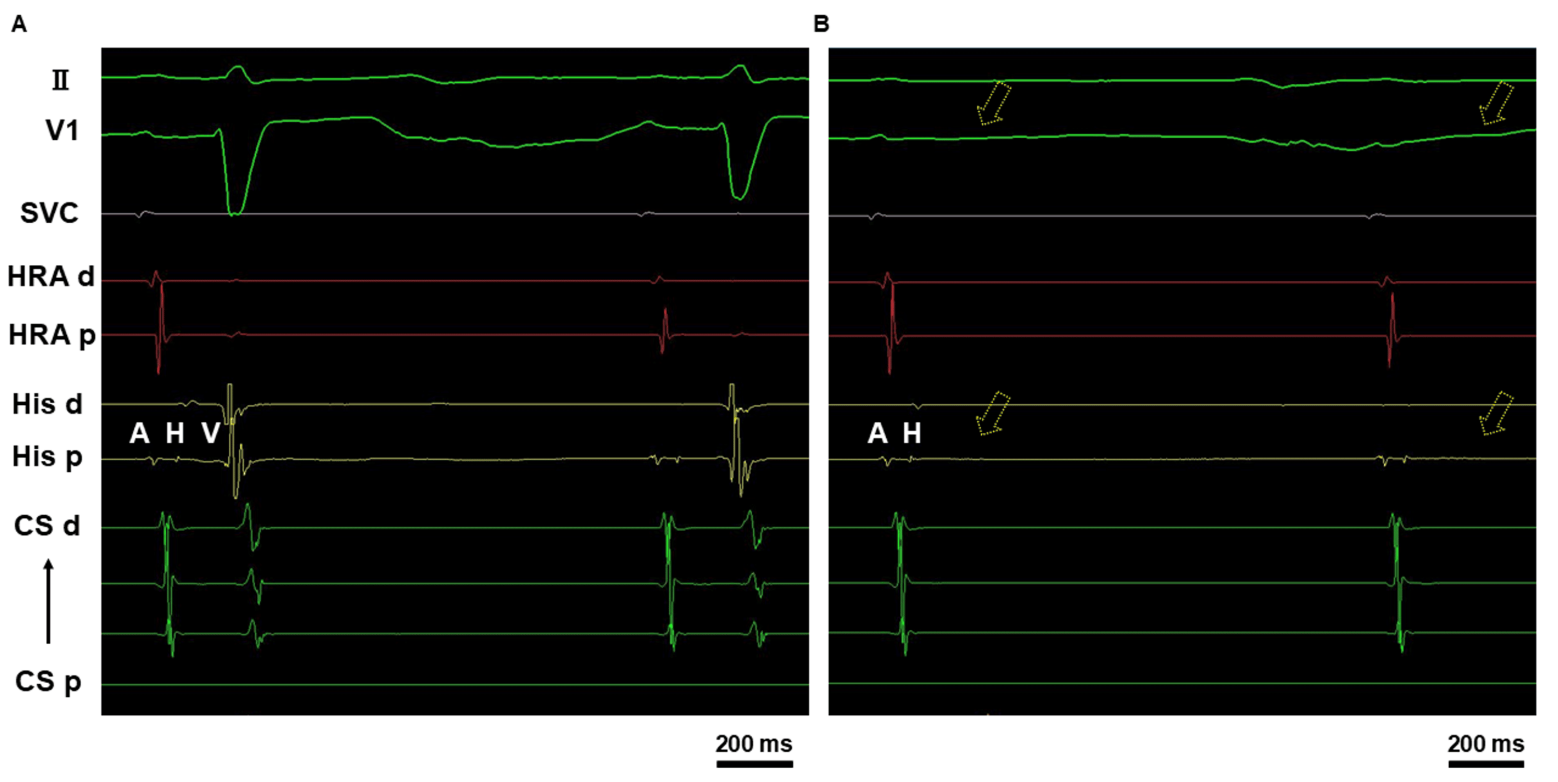
Electrophysiological study Baseline electrophysiological study showing sinus cycle length of 1000 ms, AH interval of 71 ms, and HV interval markedly prolonged to 100 ms (normal range: 35–45 ms, A). Transient HV block occurs easily with catheter stimulation (yellow arrow, B) AH = Atrio-His; HV = His-Ventricular

Three months later, the patient was readmitted to our hospital for re-ablation. ECG and transthoracic echocardiogram (TTE) showed resolution of the negative T waves and improvement in left ventricular (LV) wall motion. On the second hospital day, electrophysiologic study (EPS) and catheter ablation were performed. A noninvasive transcutaneous pacemaker was applied before the procedure. An echo-guided right internal jugular vein puncture was performed, and a guide wire was inserted into the heart under fluoroscopic guidance. During the EPS, a right atrium-coronary sinus (RA-CS) catheter was placed into the RA and CS through the right internal jugular vein. An electrophysiological examination catheter was positioned in the His area via the right femoral vein. At baseline, the sinus cycle length was 1000 ms, the atrio-His (AH) interval was 71 ms, and the His-ventricular (HV) interval was markedly prolonged to 100 ms (normal range: 35-45 ms, Figure 5A). Transient HV block was easily induced with catheter stimulation, making further examination challenging (Figure 5B). A Wenckebach rate of 120 bpm was observed under sedation. Pulmonary vein isolation, posterior wall isolation, and superior vena cava isolation were performed. Postoperatively, amiodarone 100 mg/day was discontinued. Bisoprolol 1.25 mg/day was continued to prevent the recurrence of atrial fibrillation. No complications occurred, and the patient was discharged home on day 5. No episodes of paroxysmal AVB have been observed since then.

## Discussion

LBBB is often associated with organic heart disease but can be an incidental finding in asymptomatic individuals with apparently normal hearts [3]. Hardarson et al. reported the prevalence of LBBB at 0.43% in middle-aged men and 0.28% in women [4]. In older adult patients with chronic conditions, the precise mechanisms underlying the conduction block are typically unclear, though microscopic remodeling and myocardial fibrosis are believed to play a role [3]. In this case, the patient was diagnosed with CLBBB nine years ago. However, echocardiography at the time revealed normal left ventricular wall motion, age-appropriate diastolic dysfunction, and no evidence of overt cardiomyopathy. Due to the patient’s advanced age and renal dysfunction, additional tests such as cardiac computed tomography or magnetic resonance imaging were not performed.

Padanilam et al. reported that all patients with preexisting LBBB and an r wave ≥ 1 mm in lead V1 developed RBBB, not paroxysmal AVB, due to catheter trauma to the RBB [5]. In this case, although the ECG showed LBBB with an r wave of 4 mm in lead V1, the patient developed paroxysmal AVB rather than RBBB. This finding represents a novel observation that deviates from previous reports. Additionally, the EPS revealed a markedly prolonged HV interval of 100 ms, despite no PQ interval prolongation on the ECG. Yamamoto et al. reported that rapid-rate ventricular pacing can induce overdrive suppression lasting approximately 10 s in patients with His bundle block or HV block [6]. Given that our patient had an HV block, it is plausible that the cause of paroxysmal AVB was mechanical trauma, and overdrive suppression caused by guide wire stimulation contributed to the failure of ventricular escaped beat.

Procedural sedation with dexmedetomidine may assure safety and patient immobility during AF ablation [7]. On the other hand, previous reports have highlighted that one of the most serious adverse effects of dexmedetomidine is cardiac arrest [8,9]. Increased concentrations of dexmedetomidine in humans have been associated with progressive sedation, analgesia, and decreases in heart rate and cardiac output [10]. In the present case, dexmedetomidine was administered at a rate of 6 µg/kg/min as a loading dose for 10 min. We speculate that the sedation may have contributed to the failure of ventricular escape beats. Case-specific caution is warranted regarding the rate of dexmedetomidine administration. Moreover, we hypothesize that the use of antiarrhythmic drugs, such as amiodarone and bisoprolol, may have played a role in the failure of ventricular escape beats.

To the best of our knowledge, this is the first reported case of cardiac arrest due to inadequate ventricular escape beats during catheter ablation, followed by Takotsubo cardiomyopathy during CPR. An association between adrenaline administration and Takotsubo cardiomyopathy has been reported [11,12]. We speculate that sedation with dexmedetomidine, the use of antiarrhythmic drugs, and overdrive suppression may contribute to the failure of ventricular escape beats. During the re-ablation procedure, a noninvasive transcutaneous pacemaker and fluoroscopic guide wire manipulation were employed to prevent complications. In patients with preexisting LBBB, guide wire stimulation during catheter ablation can potentially lead to cardiac arrest.

Moreover, the risk of catheter-induced paroxysmal AVB is considered to be higher during myocardial biopsy. In recent years, the number of myocardial biopsies has increased in patients with suspected transthyretin-type cardiac amyloidosis and preexisting CLBBB in Japan, particularly due to the prescription of tafamidis [13,14]. We recommend that a noninvasive transcutaneous pacemaker be prepared and fluoroscopic guide wire manipulation be employed to mitigate risk. The limitations of current preoperative risk assessment for catheter ablation in patients with preexisting LBBB should be carefully considered.

## Conclusions

In catheter ablation procedures for patients with LBBB, guide wire stimulation can potentially lead to cardiac arrest. Therefore, it is crucial to have a noninvasive transcutaneous pacemaker readily available and to use fluoroscopic guidance for guide wire manipulation to prevent complications.
